# Game location effect in game-related statistics and pre-shot combination differences between winners and losers during the basketball ACB COVID-19 season

**DOI:** 10.1371/journal.pone.0303908

**Published:** 2024-07-12

**Authors:** Álvaro Bustamante-Sánchez, Sergio L. Jiménez-Saiz

**Affiliations:** 1 Universidad Europea de Madrid, Faculty of Sport Sciences, Villaviciosa de Odón, Madrid, Spain; 2 Universidad Rey Juan Carlos, Sport Sciences Research Centre, Fuenlabrada, Madrid, Spain; UMIT TIROL Private University for Health Sciences and Technology GmbH: UMIT TIROL Private Universitat fur Gesundheitswissenschaften und -technologie GmbH, GERMANY

## Abstract

The aim of this study was to assess pre-shot combinations and game-location influence, together with the traditional game-related statistics to evaluate their impact in the performance of the teams in the Asociación de Clubs de Baloncesto (ACB) Spanish basketball league. The COVID-19 season was a great opportunity to better analyse the effect of game-location among ACB-teams to evaluate the differences between winners and losers. A retrospective cross-sectional study of the 2019–2020 ACB season (137 games of the regular season and 33 games of the final stage) was carried out. Game-related statistics were gathered from InStat (https://basketball.instatscout.com/). To evaluate the key performance indicators in this context, a discriminant analysis together with a binary logistic regression were run. The main results revealed that specific variables (normalized per minute played), such as points, field-goal percentage, field goals made, defensive rebounds, assists, and contested field goals made, played a crucial role in classifying winners and losers (p<0.05). Defensive rebounds (0.29±0.05 vs 0.24±0.04, p < 0.001), and assists (0.23±0.05 vs 0.19±0.04, p = 0.042), were key performance indicators for home-court winners. Defensive rebounds (0.29±0.05 vs 0.25±0.04, p < 0.001), and field-goal percentage (48.2±5.31 vs 42.7±5.95, p = 0.009) were key performance variables for away-court winners. Neutral-court winners had better isolation efficiency (49.5±31.6 vs 33.3±31.4, p < 0.05) and contested shot percentage (48.2±6.02 vs 44.8±5.34, p < 0.05) in the context of neutral-court tournaments. Uncontested shots and pick-and-roll efficiency seem not to be so important in the ACB when compared to the (National Basketball Association) NBA. Teams should look for players who assist to good shooters, as well as the presence of specialized players proficient in defensive rebounding. In the context of neutral-court tournaments, the importance of isolations and contested-shot performance is critical.

## Introduction

Traditionally, basketball performance has been assessed through game-related statistics, which evaluates the outcome of each possession until a new possession starts [1–4], although these outcomes usually depend on previous team structures and pre-shot combinations which influence success [5].

The collective behaviours which precede the outcome of a basketball possession help to better understand the playing structures in which basketball coaches should focus to maximize their team performance [6, 7]. Pick and rolls seem to remain the most used structure to end a play because they are the best offensive structure to gain a spatial advantage in a short period of time [8], but there are other pre-shot combinations that are highly linked to game-related statistics that have been traditionally considered important to secure a winning game outcome. For instance, defensive rebound is one of the most important feature of the game to control [3, 5, 9], and its importance seems to be related to the opportunity to make a transition [1, 2] and to avoid a second chance for the opponents through an offensive rebound [10]. Similarly, the field-goal percentage is a key factor to win a game [9], especially when the number of possessions is similar between teams [11]. This factor is related to performance in uncontested shots, in which elite players are supposed to score [5]. These opportunities are usually made after a successful space creation dynamic which usually defines a good collective offensive behaviour [5].

Contested shots are supposed to be part of the success of a team in a game because the opponents should defend well to avoid open shots. Not only the defender who guards the player with the ball can contest the shot during a 1on1 situation, but also the first player to reach the offensive player according to each team defensive structure. This behaviour also applies to any zone defensive strategy that usually tend to guard the ball handler as it was an 1on1 situation, while the other 4 players defend an area of the court with the aim to collapse the space of the opponents [12]. Because of this dynamic, many teams need players who play well on isolations to overcome a good defensive strategy, especially during the last seconds of each possession [12, 13].

Game location is also a variable to be considered when analysing the behaviour and performance of a team [14], because it may affect the mental and physical aspects due to a lack of time to recover before the game [15, 16], or because of the influence of the fans when the team is playing away [17]. Game location also affects differently depending on each playing position, with the guards being more efficient at home games, the forwards at away games, and the centers being the less influenced players by game location [18]. Although the game location seems to favour the home teams [19–21], its influence needs to be better explored with the inclusion of competitive games played in a neutral court, to include a control group among the traditional home versus away game-location comparisons [5, 22]. This study is significant and novel in the context of Spanish professional basketball, because it addresses the need of a control group of competitive games played in a neutral-court during the season to better assess the game-location influence.

The 2019–20 season was different from the others because of the COVID-19 pandemic and therefore the Asociación de Clubs de Baloncesto (ACB) competition (i.e., first basketball league in Spain) had to be completed in a neutral court at the end of the season. This situation brought the opportunity to analyse and compare the effect of playing home, away, and in a neutral court to better assess the game-related statistics and pre-shot combinations of Spanish professional basketball teams.

Thus, the aim of this study was to assess differences between losers and winners, considering their game-related statistics and pre-shooting combinations, including pick-and-roll performance. We also aimed to evaluate the effect of the game-location (neutral, home, away) to understand the performance indicators that influence better the outcome of a game. According to previous research, we hypothesized that winners would grab more rebounds and have better field-goal percentages; would perform better in isolations and transitions; and would have more contested shots with a more effective pick-and-roll performance.

## Materials and methods

In this section we will explain the design, the sample, the procedure, and the statistical analysis that were performed to reach the aim of the study.

### Design

To analyse the performance indicators related to the game result and game location, a retrospective cross-sectional study of the 2019–2020 Spanish ACB League was conducted. The study was designed in compliance with the recommendations for clinical research of the Declaration of Helsinki and approved by the Ethics Committee of the University (CIPI/19/095). The need for individual consent was waived since game data were collected from a commercially accessible provider (InStat, Moscow, Russia). The data were accessed for research purposes from September 16, 2021, to October 10, 2021.

### Sample

170 games were analysed during the 2019–2020 ACB season. To assess the performance indicators in the three different locations (playing at home n = 137, away n = 137 or in a neutral court n = 33). Our selection criterion was to include in the sample all the games played by the 12 teams that participated in the ACB final stage in Valencia to compare their performance when they played at home, away, and in a neutral court during the final stage. We therefore discarded the 6 teams that were part of the 2019–2020 ACB league which did not play any official game in a neutral court.

### Procedure

To gather the data, Instat basketball reports (Instat, Moscow, Russia) were used. For each game, performance indicators were classified into two groups of analysis, depending on the game result: winners and losers. These groups were subsequently categorised in subgroups depending on the game location: home, away, or in a neutral court. We used Instat dashboard filters to select all the performance indicators for the teams that were included in the sample, and to classify them into different study groups. Once the data was gathered, we exported it to an Excel file and to SPSS format to perform the statistical analysis. According to previous studies [5, 13, 21, 23–26], we selected the following performance indicators:

- Game-related statistics: possessions, points per possession, points, field goals made, field goals missed, field goals percentage, two-point field goals made, two-point field goals missed, two-point field goals percentage, three-point field goals made, three-point field goals missed, three-point field goals percentage, free throws made, free throws missed, free throws percentage, rebounds, offensive rebounds, defensive rebounds, assists, steals, turnovers, blocks, fouls, fouls drawn.
- Pre-shot combinations: transitions made, transitions missed, percentage of offensive transitions, catch and shoot made, catch and shoot missed, catch and shot percentage, catch and drive made, catch and drive missed, catch and drive percentage, post up made, post up missed, post up percentage, isolations made, isolations missed, isolation percentage, hand-off made, hand-off attempted, hand-off percentage, cuts made, cuts missed, cuts percentage, drives made, drives attempted, drives percentage.
- Pick and rolls (PnR), Pick and pops (PnP), and defensive parameters: PnR handlers made, PnR handlers missed, PnR handlers percentage, PnR rollers made, PnR rollers missed, PnR rollers percentage, PnP made, PnP missed, PnP percentage, uncontested field goals made, uncontested field goals missed, uncontested field goals percentage, contested field goals made, contested field goals missed, contested field goals percentage.

Table 1 shows the action definitions that InStat provides.

**Table 1:** Action definitions by InStat.

Action	Definition
Possession	Ball possession by player/team is a state when player/team controls the ball. Possession lasts since throw-in/catching the ball till FG/losing the ball. Ball possessions are manually counted by InStat.
Field goals attempted	Player’s action aimed at scoring the ball into the opponent’s basket. Shots percentage–is the ratio of shots made to shots attempted.
2-pt field goals	Field goal attempt made inside of three-point line or when player (his foot) touches it.
3-pt field goals	Field goal attempt in a basketball game made from beyond the three-point line.
Free throws	Shot from a free-throw line which is awarded after a foul on the shooter by the opposing team or after going over foul limit.
Assist	Pass to a teammate that directly leads to a made field goal.
Block	Defensive action when a defensive player legally deflects a field goal attempt from an offensive player by “covering” it with hand(s).
Foul	Breach of the rules. There are personal, technical, unsportsmanlike, flagrant fouls and team fouls. The game is stopped after the foul and followed by free-throw or an inbounds situation.
Foul drawn	A foul made by the opponents. The game is stopped after the foul and followed by free-throw or an inbounds situation.
Rebound	Retrieving the ball bouncing off the rim or backboard or after a missed shot. If player from a defending team regains the ball this is—“defensive rebound”, if the ball is recovered by the offensive side—“offensive rebound”.
Turnover	Action resulted in losing the ball by the team on offense to the opposing team.
Steal	Action when defending player causes turnover either by taking away the ball or stealing opponent’s pass.
Contested/Uncontested shot	If there is opponent between the rim and a shooter—it’s a contested shot. If there’s no opponent—uncontested.
Transitions	Ball possessions which start at team’s backcourt and lasts more than 4 and less than 8 seconds.
Pick and roll	An offensive play in which a player sets a screen (pick) for a teammate handling the ball and then slips behind the defender (rolls). There are two types: Pick and roll handler—when a ballhandler makes a shot attempt; Pick and roll roller—when a screener makes a shot.
Isolation	An offensive play when a team gives the ball handler room to play one-on-one against his opponent.
Catch and shoot	Play which finished by a jumping shot at least 3 meters from the rim by a player who controlled the ball less than 2 seconds or didn’t dribble.
Post up	Play when an offensive player receives the ball within 4,5 meters with his back to the basket and making a shot attempt.
Hand off	A play when a ball handler is squeezed between his opponent and teammate and passes the ball to his teammate for a shot attempt.
Cut	A play when a player attempting to shoot receives the ball while running towards the rim. It also includes screens, fast breaks, and situations when a player gets open at the rim.
Catch and drive	Play which finished by a shot at 3 meters or less from the rim by a player who dribbled the ball after a pass.
Drive	Play which finished by a shot at 3 meters or less from the rim by a player who dribbled.

To assess the validity and reliability of the data, a subsample of 10 games were randomly selected, and were analysed by two experienced coaches. Intra-class correlations were very good (Kappa values > 0.81).

### Statistical analysis

Normality assumptions were checked with the Kolmogorov-Smirnov test. Homoscedasticity assumptions were checked with the Levene test. Descriptive statistics were presented as mean and standard deviation. A factorial ANOVA test was used to compare the effect of the game result (win, lose), the effect of the game location (neutral-court, home-court, and away-court), and the interaction between the result and the game location. A Bonferroni post hoc test was used to analyse pairwise comparisons. The level of significance for all the comparisons was set at p< 0.05. The effect size was assessed by the eta squared value (η^2^) as specified in previous research [27]. A discriminant analysis was run to assess the main performance indicators that discriminate between the teams (winners and losers). We used the leave-one-out method to ensure the cross-validation of data, which generates the discriminant analysis n times taking out one of the games of the samples [3, 28]. We looked at the structured coefficients greater than |0.30| to select the most important variables [3, 5]. We considered the aforementioned variables to run a binary logistic regression model so we could assess the coefficients that most influence the corresponding explanatory variable, with their 95% confidence intervals (CI) [5, 29]. The IBM SPSS statistical package version 21.0 for Windows (IBM Corp., Armonk, NY) was used to analyse the data.

## Results

Table 2 shows the performance differences of game-related statistics (per possession) between winners and losers. Winners had better results in field-goals made, field-goals percentage, and rebounds. Neutral-court winners had more steals, free-throws made and attempted, and fewer turnovers. Home-court winners had more 2-point field-goals made, more 3-point field-goals made, more assists, better 2-point and 3-point percentages, 3-point field-goal attempts, and fewer fouls. Away-court winners had more 2-point field-goals made, more assists, better 2-point and 3-point field-goals percentages, and fewer turnovers and fouls. The effect size for all variables was small.

**Table 2:** Results of game-related statistics (per possession).

	Neutral				Home				Guest						
	Lost		Win		Lost		Win		Lost		Win		Game location and result		
	M	SD	M	SD	M	SD	M	SD	M	SD	M	SD	F	p	η^2^
Possessions	84.8	5.45	84.3	4.35	83.5	5.28	83.6	4.39	84.0	5.84	83.5	4.34	0.122	0.885	0.001
Points	2.22	0.48	2.24	0.56	2.17	0.53	2.32	0.48	2.29	0.55	2.33	0.48	0.496	0.609	0.003
FG made	0.34*	0.03	0.37*	0.04	0.33*	0.04	0.37*	0.04	0.32*	0.04	0.36*	0.04	0.599	0.550	0.004
FG missed	0.52	0.05	0.53	0.07	0.53	0.07	0.51	0.06	0.53*	0.07	0.50*	0.06	1.844	0.160	0.011
FG %	45.1*	4.29	47.9*	6.07	43.2*	5.98	48.9*	5.78	42.7*	5.95	48.2*	5.31	1.665	0.191	0.010
2P FG made	0.22	0.03	0.24	0.04	0.23*	0.04	0.25*	0.05	0.22*	0.04	0.24*	0.05	0.113	0.894	0.001
2P FG missed	0.20	0.04	0.21	0.05	0.23	0.06	0.21	0.06	0.22*	0.05	0.20*	0.05	1.867	0.156	0.011
2P FG %	53.7	5.93	54.7	8.15	50.8*	9.26	55.5*	7.59	50.7*	9.08	55.6*	7.40	1.497	0.225	0.009
3P FG made	0.11	0.04	0.12	0.03	0.09*	0.03	0.11*	0.04	0.10	0.03	0.11	0.04	0.290	0.749	0.002
3P FG missed	0.21	0.04	0.20	0.05	0.21*	0.05	0.19*	0.05	0.22*	0.04	0.19*	0.04	0.410	0.664	0.002
3P FG %	33.8	3.43	38.3	8.95	32.0*	10.8	38.7*	8.99	31.3*	9.79	36.9*	9.31	0.264	0.768	0.002
FT made	0.14*	0.05	0.17*	0.06	0.17	0.06	0.18	0.06	0.16	0.06	0.17	0.07	0.874	0.418	0.005
FT missed	0.04‡‡‡	0.02	0.05	0.02	0.05	0.03	0.06	0.04	0.06‡	0.03	0.05	0.03	1.191	0.305	0.007
FT %	77.2	13.2	78.0	10.8	75.7	11.5	76.2	12.2	74.4	12.2	76.3	10.0	0.131	0.877	0.001
Rebounds	0.35*	0.05	0.38*	0.05	0.36*	0.06	0.41*	0.06	0.37*	0.05	0.40*	0.06	0.814	0.444	0.005
Offensive Rebounds	0.08 ‡‡, ‡‡‡	0.03	0.10	0.03	0.11 ‡	0.04	0.11	0.03	0.11 ‡	0.04	0.10	0.04	2.097	0.124	0.012
Defensive Rebounds	0.26	0.03	0.28	0.04	0.24	0.04	0.29	0.05	0.25	0.04	0.29	0.04	2.279	0.104	0.013
Assists	0.21	0.04	0.23	0.04	0.19*	0.04	0.23*	0.04	0.19*	0.05	0.22*	0.05	0.959	0.384	0.006
Steals	0.07*	0.02	0.09*	0.03	0.07	0.02	0.08	0.03	0.07	0.02	0.08	0.03	1.331	0.266	0.008
Turnovers	0.16*	0.03	0.13*	0.03	0.15	0.04	0.14	0.03	0.16*	0.03	0.15*	0.03	1.728	0.179	0.010
Blocks	0.02	0.02	0.02	0.02	0.03	0.02	0.03	0.01	0.02	0.02	0.03	0.02	0.235	0.791	0.001
Fouls	0.23	0.04	0.21	0.03	0.23*	0.03	0.22*	0.03	0.24*	0.04	0.23*	0.03	0.031	0.970	0.000
Fouls drawn	0.21‡‡	0.03	0.23‡‡	0.04	0.24‡	0.03	0.25‡	0.03	0.23	0.03	0.24	0.03	0.186	0.830	0.001

*Note*. M: Mean. SD: Standard Deviation. F: Fisher-Snedecor test. η2: Partial eta squared. FG = Field Goals. 2P = Two-point. 3P = Three-point. FT = Free throw.

‡ Difference with neutral court (p<0.05)

‡‡ Difference with home court (p<0.05)

‡‡‡ Difference with guest court (p<0.05)

*Differences between win/lost groups (p<0.05).

Table 3 shows the performance differences in pre-shot combinations (per possession) between winning and losing teams. Neutral-court winners had more post-up missed and a better percentage of isolations. Home-court winners had better results in transitions made, catch-and-shoot made, catch-and-shoot percentage, catch-and-drive made, catch-and-drive percentage, and drives percentage, but fewer hands-off missed. Away-court winners had better results in catch-and-shoot, post-ups, posts-up percentage, cuts made, and cuts percentage, but fewer hand-offs missed. The effect size for all variables was small.

**Table 3:** Results of pre shot combinations (per possession).

	Neutral				Home				Guest						
	Lost		Win		Lost		Win		Lost		Win		Game location and result		
	M	SD	M	SD	M	SD	M	SD	M	SD	M	SD	F	p	η^2^
Transitions Missed	0.02	0.02	0.03	0.01	0.03	0.02	0.03	0.02	0.02	0.01	0.02	0.02	0.929	0.396	0.006
Transitions Made	0.04	0.02	0.04	0.02	0.03*	0.02	0.05*	0.02	0.03	0.02	0.04	0.02	2.338	0.098	0.014
Transitions %	62.7	27.5	59.0	21.3	50.9	26.2	63.7	22.4	58.5	25.1	62.6	22.4	2.624	0.074	0.015
Catch and shoot Missed	0.12	0.04	0.11	0.04	0.10	0.03	0.10	0.03	0.10	0.04	0.09	0.04	0.201	0.818	0.001
Catch and shoot Made	0.07‡	0.03	0.07	0.03	0.05*	0.02	0.07*	0.03	0.05* ‡‡‡	0.02	0.07*	0.03	1.303	0.273	0.008
Catch and shoot %	37.4	12.2	39.8	12.4	35.4*	15.0	41.6*	12.7	32.9	14.4	42.5	14.9	1.504	0.224	0.009
Catch and drive Missed	0.03	0.01	0.03	0.02	0.02	0.02	0.03	0.02	0.03	0.02	0.02	0.02	0.642	0.527	0.004
Catch and drive Made	0.02	0.01	0.02	0.01	0.01* ‡‡‡	0.01	0.02*	0.01	0.02 ‡‡	0.02	0.01	0.01	3.065	0.048	0.018
Catch and drive %	36.4	26.2	43.3	25.3	33.7*	34.9	45.4*	28.7	39.0	29.8	44.6	29.6	0.352	0.704	0.002
Post-up Missed	0.02*	0.01	0.03*	0.02	0.03	0.02	0.02	0.02	0.02	0.01	0.03	0.02	2.279	0.104	0.013
Post-up Made	0.01	0.01	0.02	0.02	0.02	0.02	0.02	0.02	0.01*	0.01	0.02*	0.01	2.071	0.128	0.012
Post-up %	42.8	28.3	39.1	26.1	41.5	34.9	43.7	27.9	39.4*	30.8	50.2*	26.2	1.553	0.213	0.009
Isolations Missed	0.03	0.02	0.03	0.02	0.02	0.02	0.02	0.02	0.03	0.02	0.02	0.02	0.225	0.798	0.001
Isolations Made	0.01	0.01	0.02	0.01	0.01	0.01	0.01	0.01	0.01	0.01	0.01	0.01	0.411	0.663	0.002
Isolations %	33.3*	31.4	49.5*	31.6	29.1	31.1	37.4	33.4	33.2	32.9	35.5	32.9	1.040	0.355	0.006
Hand-off Missed	0.02	0.02	0.02	0.02	0.02*	0.02	0.01*	0.01	0.02*	0.02	0.01*	0.01	2.017	0.135	0.012
Hand-off Made	0.01	0.01	0.01	0.01	0.01	0.01	0.01	0.01	0.01	0.01	0.01	0.01	0.101	0.904	0.001
Hand-off %	30.1	31.4	35.9	32.0	39.1	33.1	43.6	41.2	29.4	30.9	32.9	35.5	0.022	0.978	0.000
Cuts Missed	0.02	0.01	0.02	0.02	0.02	0.01	0.02‡‡‡	0.02	0.02	0.02	0.01‡‡	0.01	2.209	0.111	0.013
Cuts Made	0.03	0.02	0.03	0.02	0.03	0.01	0.03	0.02	0.03*	0.02	0.03*	0.02	1.741	0.177	0.010
Cuts %	96.1	22.6	67.0	24.2	65.4	28.4	64.2	27.5	63.4*	30.6	71.4*	22.2	1.524	0.219	0.009
Drives Missed	0.09	0.03	0.09	0.03	0.03	0.04	0.08	0.04	0.10	0.04	0.08	0.04	0.697	0.499	0.004
Drives Made	0.06	0.03	0.07	0.03	0.06	0.02	0.07	0.03	0.06	0.02	0.07	0.03	0.284	0.753	0.002
Drives %	42.4	14.8	16.0	11.7	42.9*	16.1	48.2*	15.5	42.5	15.2	47.3	13.6	0.067	0.935	0.000

*Note*. M: Mean. SD: Standard Deviation. F: Fisher-Snedecor test. η2: Partial eta squared

‡ Difference with neutral court (p<0.05)

‡‡ Difference with home court (p<0.05)

‡‡‡ Difference with guest court (p<0.05)

*Differences between win/lost groups (p<0.05).

Table 4 shows the performance differences for picks and defensive indicators (per possession) between winning and losing teams. Neutral-court winners had more contested shots made and a better percentage of contested shots. Home-court winners had better pick-and-roll-handlers percentages, better pick-and-roll-rollers percentages, better contested-shots percentages, and more contested-shot made. Away-court winners had more contested-shots made, and a better contested-shots percentage, but had fewer uncontested-shot made. The effect size for all variables was small.

**Table 4:** Results of picks and defensive indicators (per possession).

	Neutral				Home				Guest						
	Lost		Win		Lost		Win		Lost		Win		Game location and result		
	M	SD	M	SD	M	SD	M	SD	M	SD	M	SD	F	p	η^2^
PnR handlers Missed	0.07	0.03	0.06	0.03	0.08*	0.04	0.07*	0.04	0.08	0.04	0.07	0.03	1.008	0.366	0.006
PnR handlers Made	0.04	0.02	0.04	0.02	0.05	0.02	0.05	0.03	0.04	0.02	0.05	0.02	0.094	0.911	0.001
PnR handlers %	38.2	17.9	44.4	18.8	37.7*	16.8	46.0*	20.9	39.1	18.1	40.8	16.5	1.017	0.363	0.006
PnR rollers Missed	0.07‡‡	0.03	0.07	0.03	0.10‡,*	0.04	0.08*	0.04	0.09	0.04	0.09	0.04	1.564	0.211	0.009
PnR rollers Made	0.06	0.02	0.07	0.03	0.07	0.02	0.07	0.03	0.06	0.02	0.07	0.03	0.349	0.706	0.002
PnR rollers %	43.0	15.0	49.9	15.4	41.9*	12.3	51.0* ‡‡‡	18.6	42.7	16.2	43.4‡‡	12.6	2.369	0.095	0.014
PnP Missed	0.01	0.02	0.01	0.01	0.00	0.00	0.01	0.01	0.01	0.01	0.01	0.01	0.347	0.707	0.002
PnP Made	0.01	0.01	0.01	0.01	0.01	0.01	0.01	0.01	0.01	0.01	0.01	0.01	0.260	0.771	0.002
PnP %	20.3	31.2	26.7	31.6	27.9	38.6	28.3	37.9	26.4	39.4	28.3	35.6	0.141	0.869	0.001
Uncontested Missed	0.09	0.06	0.09	0.05	0.10	0.06	0.09	0.06	0.11*	0.07	0.08*	0.06	1.132	0.324	0.007
Uncontested Made	0.06	0.03	0.05	0.03	0.05	0.03	0.07	0.04	0.06	0.03	0.06	0.03	1.453	0.235	0.009
Uncontested %	41.4	14.0	37.1	16.8	38.0	16.2	40.4	17.1	35.5	15.2	39.5	19.1	1.388	0.251	0.008
Contested Missed	0.32	0.05	0.31	0.07	0.33*	0.07	0.30*	0.07	0.33	0.07	0.31	0.07	0.743	0.476	0.004
Contested Made	0.26*	0.04	0.29*	0.04	0.25*	0.04	0.27*	0.05	0.24*	0.05	0.28*	0.04	0.760	0.469	0.005
Contested %	44.8*	5.34	48.2*	6.02	43.4*	5.80	48.2*	7.11	43.0*	7.90	48.4*	6.25	0.484	0.617	0.003

*Note*. M: Mean. SD: Standard Deviation. F: Fisher-Snedecor test. η2: Partial eta squared. PnR = Pick and roll; PnP = Pick and pop

‡ Difference with neutral court (p<0.05)

‡‡ Difference with home court (p<0.05)

‡‡‡ Difference with guest court (p<0.05)

*Differences between win/lost groups (p<0.05).

Table 5 shows the discriminant analysis, which was statistically significant (p < 0.05), and it could correctly classify 86.8% of the cases, as an average of the n iterations that were specified in the statistical analysis section. The model found these discriminants to classify winners and losers: points (SC = 0.511), field-goal percentage (SC = 0.424), field goals made (SC = 0.419), defensive rebounds (SC = 0.367), assists (SC = 0.332), contested field-goals made (SC = 0.304).

**Table 5:** Discriminant function structure coefficients (SC) and tests of statistical significance (86,8% correctly classified ‡).

**Game statistic**	**SC**	**Game statistic**	**SC**
Points	0.511*	Drives missed	-0.114
FG %	0.424*	PnR Rollers missed	-0.114
FG Made	0.419*	Post-up made	0.113
Defensive Rebounds	0.367*	Catch and shot missed	-0.110
Assists	0.332*	PnR Rollers made	0.103
Contested FG made	0.304*	Drives made	0.093
Rebounds	0.297	2PT FG missed	0.089
3PT FG %	0.294	Cuts made	0.084
3PT FG missed	-0.281	FT missed	0.078
2PT FG made	0.263	Handoffs made	-0.074
2PT FG %	0.234	Catch and drive missed	-0.063
Handoff missed	-0.214	Isolations made	0.062
Turnovers	-0.210	Blocks made	0.054
Catch and Shoot made	0.195	Uncontested FG made	0.042
3PT FG made	0.175	Cuts missed	0.041
Transitions made	0.169	PnP Missed	0.040
Fouls	-0.169	FT %	0.039
FG missed	-0.153	PnR Handlers made	0.039
FT made	0.153	Catch and drive made	0.029
Uncontested FG missed	-0.144	Isolations missed	-0.029
Steals	0.140	Post up missed	0.028
Fouls drawn	0.135	Transitions missed	-0.017
PnR Handlers missed	-0.133	PnP made	0.009
Contested FG missed	-0.131	Offensive rebounds	0.008

Note

‡ Cross-validation method: leave-one-out. FG = Field Goals. 2P = Two-point. 3P = Three-point. FT = Free throw PnR = Pick and roll; PnP = Pick and pop

* SC discriminant value > |0.30|.

Table 6 shows the analysis of the binary logistic regression. Defensive rebounds (p < 0.001; OR = 3.044E13), and assists (p = 0.042; OR = 353289.356), were statistically significant for home-court winners. Defensive rebounds (p < 0.001; OR = 2.676E12), and field-goal percentage (p = 0.009; OR = 1.198) were statistically significant for away-court winners. No variables were statistically significantly associated with winning for neutral-court teams.

**Table 6:** Binary logistic regression values.

Variables	B	P	Exp(B)	95% C.I. EXP(B)	
				Lower	Upper
**Game location: neutral**					
FG Made	13.155	0.298	516832.809	0.000	3.031E16
FG %	-0.31	0.698	0.969	0.827	1.135
Defensive Rebounds	13.227	0.059	583450.242	0.600	5.673E11
Assists	3.188	0.687	24.243	0.000	1.315E8
Contested FG made	10.857	0.199	51900.991	0.003	8.054E11
Constant	-10.596	0.003	0.000		
**Game location: home**					
FG Made	20.938	0.053	1.239E9	0.731	2.102E18
FG %	0.092	0.168	1.097	0.962	1.250
Defensive Rebounds	31.047	0.000	3.044E13	4.538E7	2.041E19
Assists	12.775	0.042	353289.356	1.614	7.731E10
Contested FG made	-1.499	0.814	0.223	0.000	58711.222
Constant	-21.471	0.000	0.000		
**Game location: away**					
FG Made	7.812	0.412	2469.806	0.000	3.113E11
FG %	0.181	0.009	1.198	1.046	1.372
Defensive Rebounds	28.615	0.000	2.676E12	1.387E7	5.165E17
Assists	1.348	0.796	3.849	0.000	103587.861
Contested FG made	1.403	0.812	4.067	0.000	424959.608
Constant	-19.458	0.000	0.000		

*Note*. FG = Field Goals. 3P = Three-point.

## Discussion

The aim of this study was to assess differences between losers and winners, considering their game-related statistics and pre-shooting combinations, including pick-and-roll performance. We also aimed to evaluate the effect of the game-location (neutral, home, away) to understand the performance indicators that influence better the outcome of a game. The first hypothesis was that winners would grab more rebounds and have better field-goal percentages; the second hypothesis was that winners would perform better in isolations and transitions; and the third hypothesis was that winners would have more contested shots with a more effective pick-and-roll performance.

Not only the winners had better field-goal percentages and rebounds, but also, they were identified as key factors in the discriminant analysis, including assists and defensive rebounds too. Thus, the first hypothesis can be accepted. The importance of the field-goals, in the National Basketball Association (NBA) [5, 9, 30], and in the ACB league [31, 32], has been previously reported, since scoring has a direct influence in the game outcome. Moreover, this study highlights the importance of maintaining the ball and to obtain more possessions through more steals and fewer turnovers, together with more free-throw attempts when playing in a neutral court. The value of the assists to have more odds to win a basketball game has been traditionally defined in previous studies in Europe [20, 32] and in the NBA league [5, 33]. This fact should be related to a positive end of a possession (because an assist implies a basket) and a greater cohesion of the teammates to share the ball if an advantage has been created when the offensive players outnumber their opponents [34]. However, there is still some controversy about its importance by itself, or just because it is linked to a successful possession, because some spatial or numeric advantages could also be successfully resolved by the ball-handler if they find the opportunity to end the possession through an easy basket instead of a pass to assist a teammate [5, 35]. Notwithstanding, according to our results, assists are a key factor for both winning at home and away, but it is not so critical when playing on a neutral court. This issue has been argued previously suggesting that some variables are largely impacted by fans’ support [19]. The importance of rebounding seems to remain unaltered during the evolution of this sport, probably because its control produces a second chance to score (in offense), or the possibility to start a fast break, to get the possession of the ball again to build a potential scoring opportunity, and to deny the possibility of scoring by the opponents (in defence) [10, 36].

Considering the pre-shot combinations, the second hypothesis was partially accepted since home-team winners were better at transitions, and neutral-court winners were better at isolations. The catch and shoot performance was a key factor for success in winners both playing home and away and supports the information provided above about the importance of field-goals accuracy for winning in basketball, not only at the professional level in the NBA [5, 9] and the ACB leagues [13, 32], but also in the National Collegiate Athletic Association (NCAA) level [14]. However, this study adds some insights about the best type of shots that discriminate between winners and losers depending on the home-advantage effect. Home-court winners had better percentages in catch and drive and drive actions, which are typically actions taken by perimeter players. However, away-court winners had better percentages in post-up actions and cuts, which are usually actions that require to shoot very close to the basket, and that could be related to a need for a more physical playing style when playing away, plus a better accuracy in technical actions when playing at home. These findings are related to the defensive systems used by home and away teams, modifying the open spaces and tactical behaviours that may generate a decrease/increase in scoring opportunities [37].

Contested field-goals made was a discriminant factor for winners, and also winners had better contested-shot efficacy in all kinds of courts (neutral, home, and away). Home-court winners had better efficacy in pick-and-roll situations, both finishing with the ball handler and the rollers. Thus, the third hypothesis was partially fulfilled. The importance of pick-and-roll structures has been growing in recent years since this action provides a quick spatial advantage for the offensive team [7, 8, 38]. However, there is a difference between this study in the ACB league and previous research in the NBA [5], in which all teams had better percentages in pick-and-roll situations, independently of their game location. Apparently, this feature is not so strong in the ACB league because of the different defensive rules that apply to each competition. The NBA has the defensive three-second violation rule (illegal defence) that does not allow a defensive player to stay in the lane more than 3 consecutive seconds while not actively guarding an opponent, while this rule does not apply in the ACB league. This rule suggest better opportunities in the ACB league than in the NBA to contain pick-and-roll situations through defensive helps in the lane [11]. Field-goal percentage relies on accuracy for both contested and uncontested shots, although uncontested shots missed are a key factor in case the team is playing away. This study presented different results when compared to a previous one in the NBA [5], in which uncontested shots were key factors to win the game. As we mentioned before, the different defensive rules that apply in both leagues could be the main factor for these results that supports the importance of contested shots in the ACB league. Besides, there could be an influence of (i) a more structured defensive rotation to contest open shots [39]; or (ii) more space to defend and better athleticism in the NBA [40].

In the context of pandemic-affected seasons or neutral-court tournaments (i.e. Spanish basketball Cup, Euroleague Final Four, etc.), this research deepens the knowledge about the key performance indicators that are crucial for a win outcome. The importance of rebounding seems to be unaltered when compared to the standard home-team vs away-team game [5, 10, 36]. However, one of the strengths of our research is to also consider pre-shot combinations to better understand the context in which each action is decisive for a successful game outcome. According to our results, to get a better field-goal percentage to win a game [31, 32], is especially critical when shooting contested throws in a neutral-court tournament. These results also highlight the importance to improve the number of steals and to lower the occurrences of turnovers, especially to enhance the percentage of successful isolations in neutral-court situations [5]. If we consider these results globally, teams that play in neutral courts seem to be less influenced by the game-location atmosphere to look for a better collective behaviour, so they find it more difficult to play team-oriented pre-shot combinations [5, 17, 37]. In conclusion, coaches should consider this different behaviour and remember the importance of isolations and contested-shot performance as critical to be successful in neutral-court tournaments. These results and implications could only be extrapolated to matches without spectators that may occur in the future due to the special conditions they had during COVID-19 season.

## Conclusion

In conclusion, our study sheds light on the nuanced influence of game location on performance differentials between winners and losers in the ACB COVID-19 season. The findings underscore the critical role of specific variables, including points, field-goal percentage, field goals made, defensive rebounds, assists, and contested field goals made, in distinguishing between successful and unsuccessful teams across neutral-court, home-court, and away-court settings.

Notably, our analysis reveals distinct patterns among winners depending on game location. Home-court winners exhibited heightened significance of defensive rebounds and assists, whereas away-court winners leaned more heavily on defensive rebounds and field-goal percentage as predictors of success. Intriguingly, neutral-court winners appeared to adjust their play style, favouring less team-oriented pre-shot combinations, potentially influenced by the unique game-location atmosphere. Coaches should thus consider the strategic implications of emphasizing isolations and contested shot performance in neutral-court tournaments.

Furthermore, our research highlights disparities between the ACB league and the NBA, particularly regarding the perceived importance of uncontested shots and pick-and-roll efficiency. This variance may be attributed to factors such as the three-second violation rule in the NBA, impacting defensive structures and help defence strategies differently.

Overall, our study contributes novel insights into the interplay between game location, game-related statistics, and pre-shot combinations in competitive basketball contexts, providing valuable implications for coaching strategies and player performance optimization in diverse tournament settings.

Future studies should consider the importance of the crowds in the different outcomes of neutral-court games, since not only the court, but the fans could influence each team behaviour. They should also consider the different influence that neutral game-locations may have in different competitions, levels and in women leagues and tournaments.
